# Insights Into Chemosensory Proteins From Non-Model Insects: Advances and Perspectives in the Context of Pest Management

**DOI:** 10.3389/fphys.2022.924750

**Published:** 2022-08-22

**Authors:** Paula Lizana, Ana Mutis, Andrés Quiroz, Herbert Venthur

**Affiliations:** ^1^ Programa de Doctorado en Ciencias de Recursos Naturales, Universidad de La Frontera, Temuco, Chile; ^2^ Laboratorio de Química Ecológica, Departamento de Ciencias Químicas y Recursos Naturales, Facultad de Ingeniería y Ciencias, Universidad de La Frontera, Temuco, Chile

**Keywords:** pest control, sustainability, insect olfaction, model insects, non-model insects, chemosensory proteins

## Abstract

Nowadays, insect chemosensation represents a key aspect of integrated pest management in the Anthropocene epoch. Olfaction-related proteins have been the focus of studies due to their function in vital processes, such ashost finding and reproduction behavior. Hence, most research has been based on the study of model insects, namely *Drosophila melanogaster*, *Bombyx mori* or *Tribolium castaneum*. Over the passage of time and the advance of new molecular techniques, insects considered non-models have been studied, contributing greatly to the knowledge of insect olfactory systems and enhanced pest control methods. In this review, a reference point for non-model insects is proposed and the concept of model and non-model insects is discussed. Likewise, it summarizes and discusses the progress and contribution in the olfaction field of both model and non-model insects considered pests in agriculture.

## Introduction

Among living species, insects are considered the major class with an estimated more than five million species, and an important role in the ecosystem (Stork, 2018). However, with an ever-growing global population and a subsequent intensive agriculture at a global scale, an increasing number of insect species have become a threat to the food supply. Thus, the impact of insects pests on agriculture has caused serious damage to the global economy, causing losses of 20%–40% of world food production (FAO, 2017). In that sense, it has been reported that the greatest damage caused by insects lies in direct feeding on plant parts, such as chewing leaves, piercing stems and roots (Cranshaw, 2004; Oerke, 2006).

Since insecticides emerged, chemical control has been the main strategy to fight insect pests. However, their indiscriminate use as a consequence of a high demand for food has resulted in long residual on plants as well as leaching into soil, reaching groundwater and affecting soil nutritional quality, threatening the environment and living beings (Peralta et al., 1994; Arora and Sahni, 2016; Md Meftaul et al., 2020). It is believed that the inherent toxicity of insecticides has generated serious issues for human health. This is manifest in neurological symptoms and some related diseases, such as Parkinson’s disease, based on direct or exposure to these chemicals (Hertzman et al., 1994; Kamel et al., 2005; Keifer and Firestone, 2007). Over the years, insects have managed to adapt to different classes insecticides resulted in developing resistance (Heisey and Norton, 2007; Bass et al., 2014; Khan and Ahmad, 2019; Mohammed et al., 2019; Attia et al., 2020; Khan et al., 2020). For instance, the intensive use of transgenic corn with the insecticidal protein Cry1F has provoked resistance in some major corn pests, the codling moth *Spodoptera frugiperda*, affecting the economy and agriculture of Puerto Rico, Brazil and the United States (Storer et al., 2010; Storer et al., 2012; Farias et al., 2014; Huang et al., 2014; Yang et al., 2016).

Alternatively, volatile chemicals already present in nature have been extensively studied for their potential role in manipulating insect behavior (i.e., semiochemicals). Particular emphasis has been placed on sex pheromones, which are volatiles usually released by a female, that can elicit strong attraction in conspecific males. Additionally, an approach called “push-pull” that interferes with the ability of insects to find hosts by using plants capable of producing attractants and repellents has been used (Hassanali et al., 2008). Thus, these behavior-based techniques are related to olfaction and employed as effective control methods in pest management, being non-toxic nor invasive for crops (Smart et al., 2014; Soroker et al., 2015).

To understand how insects smell and their resulting behaviors, the study of their olfactory physiology has arisen as a groundbreaking field of research. Insects smell mainly through their antennae, which houses specialized units called sensilla. Inside these, odorant-binding proteins (OBPs) and chemosensory proteins (CSPs) are small extracellular proteins which thought to aid in capture and transport of odorants and pheromones to the receptors (Leal, 2005; Zhou, 2010; Leal, 2013). The OBPs and CSPs play a fundamental role in the mechanism of olfaction and in the evolutionary adaptation of the olfactory system (Pelosi and Maida, 1990; Vogt et al., 1991; Vieira and Rozas, 2011; Pelosi et al., 2018; D'Onofrio et al., 2020). On the other hand, different types of transmembrane proteins are present in olfactory receptor neurons (ORNs), such as odorant receptors (ORs), gustatory receptors (GRs), ionotropic receptors (IRs) and sensory neuron membrane proteins (SNMPs) (Croset et al., 2010; Abuin et al., 2011). Altogether, these allow the transduction of chemical signals into electrical signals (Kaissling, 2013). Currently, ORs, CSPs and OBPs are being studied comprehensively to clarify their binding specificities and mechanisms. Their use as targets to identify novel potent semiochemicals can be argued in terms of failures and successes (Venthur and Zhou, 2018). Ultimately, these approaches assumed to play an important role in integrated pest management and broadening the limited options available today.

Over the last few decades, the advance in our understanding of how insects smell was based on a few species. An important milestone included the identification and synthesis of the first sex pheromone, bombykol, from the silkmoth *Bombyx mori* (Butenandt, 1959; Butenandt, 1963; Mansurova et al., 2009). Then came the advance in molecular techniques to identify OBPs as key odorant carriers (Vogt and Riddiford, 1981) and, years later, the first complete genome of an insect that was from *Drosophila melanogaster* (Adams et al., 2000). Such events made *B. mori*, *Antheraea polyphemus* and *D. melanogaster* the first identifiable model insects for research. Later, other insect species with complete genomes, such as the malaria mosquito *Anopheles gambiae* (Holt et al., 2002) and the honeybee *Apis mellifera* (Weinstock et al., 2006), have also been considered model insects for olfaction research. Nowadays, moths, namely *Manduca sexta* and *Spodoptera littoralis*, have outstanding studies related to olfactory proteins through next generation sequencing and functional assays (Grosse-Wilde et al., 2011; de Fouchier et al., 2017).

Considering the significant advance in insect research, we believe that there is no clear definition of what makes an insect either a model or non-model. Likewise, we believe that there are insects that have not been sufficiently considered, which have outstanding features at molecular, physiological and behavioral levels that can provide key evidence for the understanding of nature, from agricultural questions to broader fields. Therefore, the present review aims to discuss the impact of studying insects with agricultural importance in the last decade as well as to identify patterns that can allow to propose a benchmark for model and non-model insects, including the nuances between both concepts. In addition, this revision provides a general perspective of the importance of insect olfaction research and hints of what future could bring for the field.

In order to retrieve information to build our datasets, a literature search was performed in the Web of Science (WOS), Scopus, PLoS and Scielo databases, mainly using the following keywords: pest control, sustainability, insect olfaction, model insects, non-model insects and chemosensory proteins. In addition, some phrases such as olfactory physiology, control methods, agriculture and chemoreception were used, thus collecting more than 300 scientific articles. These datasets can be found in Supplementary Tables S1, S2.

## What are Model and Non-Model Insects?

Although no clear definition of what either a model or non-model insect is, a few factors can be established that are common among the first identifiable model insects. For instance, *B. mori* is considered a model system for entomological studies due to its large body size, ease of rearing in laboratory, and economic importance in sericulture (Mita et al., 2004). *D. melanogaster*, on the other hand, began to be a study model because of its short generation times, ease of breeding and tolerance to relatively high population densities (Ashburner, 1989). Studies on the red flour beetle (*Tribolium castaneum*) arose because of its importance as a pest of stored grains and grain products, with a worldwide distribution, in addition to its ease of rearing, relatively short generation interval, ability to be reared in field and low maintenance (Chu et al., 2014; Kumar et al., 2018). Finally, *A. polyphemus* (similar to *B. mori*) has been extensively studied because of ease of breeding, large size and molecular mechanisms of sex pheromone action (Collins and Weast, 1961; Hall, 2015).

Considering the above, it is possible to establish common characteristics that model insects have, such as ease of rearing and size, which could have helped scientists perform early molecular biology-related methods. By contrast, non-model insects can be considered little-known species that are often specific to a geographic region. For instance, *Neotetranychus lek*, a species of the red mite family first reported in 2013 in Thailand, is believed to be endemic to Southeast Asia and a pest of cassava of agricultural importance (Flechtmann, 2013). Another example is the *Bactrocera jarvisi* fly, endemic to Australia, which is considered a pest in the territory due to the damage it causes to commercially important crops, such as mango (Peng et al., 2007; Fay, 2012). Many times, however, it depends on the study area whether an insect is considered a model or not. For example, the greater wax moth, *Galleria mellonella*, a harmful pest of beehives, can be considered a non-model insect in terms of its understanding of olfactory physiology, with some recent advances in the field (Aburto et al., 2019; Zhao et al., 2019; Lizana et al., 2020). Nevertheless, it can be a model insect due to its ease of rearing and use as hosts for the study of pathogens (Killiny, 2018; Ménard et al., 2021). Another example is the butterfly *Araschnia levana*, which can be considered a model for the study of the molecular basis and evolutionary ecology of seasonal polyphenism due to its characteristic seasonal dimorphism (Baudach and Vilcinskas, 2021). In recent years, the order Coleoptera has taken on great importance in the identification of little-known species, which can be considered models for their particularities. An example is the canola beetle *Meligethes aeneus*, considered a model to determine how agricultural practices affect pest population dynamics as well as the impact of different control strategies on the risk and speed of resistance development (Stratonovitch, et al., 2014). Another case is the potato beetle *Leptinotarsa decemlineata*, which due to its special ability to adapt to plants, environmental conditions, with particular resistance to more than 50 pesticides, can be considered a model species for agricultural pest genomics (Schoville et al., 2018). Furthermore, the number of studies of a particular species over the years may result in a species initially considered as non-model now being classified as a model species. The study of non-model insects can bring multiple advantages for researchers, such as new and original research questions and greater knowledge of pest insects of agricultural importance. This would enable the establishment of both species-specific control methods and less harmful to the environment. Moreover, the increased knowledge through research on non-model insects endemic to different geographic areas would allow the respective economic development of these areas. An example are beetles endemic to Chile, namely *Hylamorpha elegans* and *Brachysternus prasinus*. These insects are distributed in the same geographic area (central and southern part of the country) and are considered pests of economic importance due to the serious damage they cause to crops, such as wheat (*Triticum aestivum*) and red clover (*Trifolium pratense*) (Gonzalez, 1989; Artigas, 1994; Aguilera, 1995; Palma, 2004). Therefore, the study of these coleopterans would allow us to understand the sympatry present in them as well as speciation. Another example is eggplant borer *Leucinodes orbonalis* in Bangladesh, where it has been possible to establish control using pheromonal traps, thus minimizing the use of pesticides (Mazumder and Khalequzzaman, 2010).

Overall, we believe that a non-model insect has limited information on genomics and biological mechanisms, and is generally distributed in specific geographic areas.

## Role of Non-Model Insects in the Study of Olfaction

For insects, there are some key aspects for the development of their respective life cycles, such as searching for food, mate finding, a place to oviposit and even detecting a nearby predator. Interestingly, all of them depend on the olfactory system (De Bruyne and Baker, 2008). Thus, insect olfaction at perireceptor level has several types of proteins for such purposes. Soluble proteins, namely OBPs and CSPs, transport semiochemicals through the sensillar lymph to reach receptors in ORNs. These carriers function as the first filter of olfactory information for insects (Leal, 2005). Receptors (e.g., ORs, IRs and GRs), on the other hand, recognize semiochemicals, allowing signal transduction followed by a cascade of events that end in the central nervous system of insects, unleashing behavioral responses (Wicher and Marion-Poll, 2018). In particular, IRs and GRs have been a matter of interest in recent years due to their involvement in hygroreception or sugar/bitter perception, respectively (Slone et al., 2007; Jiao et al., 2008; Wanner and Robertson, 2008; Enjin et al., 2016; Kim and Wang, 2016; Knecht et al., 2017). Conversely, SNMPs have received less attention. However, these types of transmembrane proteins appear to have a key role in signal transduction, likely involved in the heteromeric complex of OR/Orco (Zhang et al., 2020). Besides these, OBPs, CSPs and ORs stand out as the most studied to date. Hence, this review will focus on these.

### Odorant Binding Proteins and Chemosensory Proteins: Key Transporters for Chemoreception

OBPs play a fundamental role in binding hydrophobic odorants, such as pheromones, plant volatiles, among others, in the pores of the sensilla and transporting them through the sensillar lymph to facilitate their solubilization (Prestwich, 1996). Thus, it is proposed that they participate in the first biochemical stage of perireceptor events in odorant capture (Jacquin-Joly and Merlin, 2004; Pelosi et al., 2005; Zhou, 2010). Defined as molecules capable of recognizing a variety of volatile compounds (Venthur et al., 2014), it has been established that OBPs act as a semiochemical solubilizers, transporters and ligand filters, mediating the activation of ORs (Fan et al., 2011). For in-depth reviews around OBPs (and CSPs too), we suggest readers revise Zhou (2010), Venthur et al. (2014), and Pelosi et al. (2018).

Insect OBPs were first identified in *A. polyphemus* (Vogt and Riddiford, 1981), adopting the nomenclature of pheromone-binding protein (PBP) for those OBPs that were male-biased in expression and that showed affinity to sex pheromones. Nowadays, this classification has remained only for Lepidopterans. An outstanding example is the PBP1 of *B. mori* (BmorPBP1), which became a study model to understand the binding mechanism of pheromone components and to expand the knowledge about how these proteins function in the olfactory system of insects (Wojtasek and Leal, 1999; Damberger et al., 2000; Horst et al., 2001; Lee et al., 2002). Outstandingly, the first three-dimensional (3D) structure for an insect OBP was BmorPBP1, showing that residue Ser59 was important for establishing hydrogen bonds with bombykol (i.e., *B. mori* sex pheromone) (Sandler et al., 2000). Later, Lautenschlager et al. (2005; 2007) were able to show that BmorPBP1 undergoes important conformational changes that depend on pH, suggesting ligand binding under neutral conditions, and release under acidic conditions (i.e., near the dendritic membrane of ORNs). Furthermore, BmorPBP1 binds and holds bombykol across different pH values, contrary to other types of odorants (Damberger et al., 2013). This certainly opened up the field to study other insect species. Currently, the 3D structure of OBPs for a diverse range of insects, such as navel orangeworm *Amyelois transitella* (Di Luccio et al., 2013), medfly *Ceratitis capitata* (Falchetto et al., 2019), aphids *Megoura viciae* and *Nasonovia ribisnigri* (Northey et al., 2016). For instance, these last structures have been used as templates to build the 3D structure of homologues OBPs for other insect species, allowing their study at bioinformatics and biochemical level. Thus, aphid *Rhopalosiphum padi*, for which OBP7 strongly bound (*E*)-β-farnesene, a repellent for *R. padi*, was supported by the use of crystal structures of *M. viciae* and *N. ribisnigri* OBPs. More recently, the crystal structure for *Epiphyas postvittana* PBP3 was reported, representing the first 3D structure of an OBP for tortricid moths, which have an enormous economic impact for agriculture worldwide (Hamiaux et al., 2020). This represents an important advance in the study of other tortricids of economic importance, such as *Lobesia botrana*, *Grapholita molesta* and *Cydia pomonella*, allowing a more precise construction of 3D models for OBPs.

CSPs, as well as OBPs, are involved in peripheral olfactory processing, and together they are the most important proteins for chemoreception due to their functions as carriers of semiochemicals (Breer et al., 1994; Calvello et al., 2003). Remarkably, early evidence not only suggested CSPs as odorant transporters, but that they were also involved in non-chemosensory functions (Qiao et al., 2013), as they have been identified in both chemosensory and non-chemosensory organs. An example of this is *Locusta migratoria*, where 17 CSPs were identified in reproductive organs (Zhou et al., 2013). Unlike OBPs that are considered to be more antenna-specific, CSPs are distributed in various insect tissues, namely legs, proboscis, thorax, etc. (Picimbon et al., 2001; Wanner et al., 2005; Foret et al., 2007). However, recent reports strongly suggest their role as carriers of semiochemicals (Li et al., 2016; Peng et al., 2017). For example, CSP1, CSP2 and CSP3 of rice leaf folder *Cnaphalocrocis medinalis* (major pest of rice plants) showed high binding affinity to host-related semiochemicals, such as terpenoids, and even sex pheromones (*Z*)-11-hexadecenyl acetate and (*Z*)-11-hexadecenal (Zeng et al., 2018). More recently, silencing of CSP4 and CSP5 in aphid *R. padi* (pest of Poaceae plants) resulted in significant reduction of octanal detection, a host plant attractant (Peng et al., 2020). In addition to the role of CSPs binding semiochemicals, their involvement in insecticide resistance has been recently reported for cotton aphid *Aphis gossypii*, a polyphagous pest with such resistance (Li et al., 2021). The authors found that CSP5 of *A. gossypii* is upregulated under insecticide treatments (imidacloprid and cypermethrin), potentially helping to decrease mortality.

The first 3D structure to be solved for CSPs was cabbage moth *Mamestra brassicae* CSP (Lartigue et al., 2002), a serious pest for *Brassica* plants. On the one hand, 3D structures for *Schistocerca gregaria* and *M. brassicae* CSPs, which have economic importance as pests, have been solved and deposited in databases (https://www.rcsb.org/). In the case of OBPs, more 3D structures are available; for example, there are 28 structures available for Lepidoptera, two structures for both Coleoptera and Hemiptera and in the case of Hemiptera, there are more than 30, though only 3 are strongly related to agricultural pests. Consequently, more structural studies can be performed for OBPs than CSPs, being a key factor to study non-model insect pests.

### Odorant Receptors and Their Importance for Understanding Insect Olfaction

In the family of membrane chemosensory receptors in insects, the so-called ORs and IRs are found. Although both receptors are responsable for chemical detection, ORs have received significant attention due to their role in capturing secondary metabolites as odorants, emitted by either host plants or conspecific insects. The latter was identified in *D. melanogaster* using its assembled genome (Adams et al., 2000), which encodes the particular 7TM for insects (Clyne et al., 1999; Gao and Chess, 1999; Vosshall et al., 1999). Then, with the identification of *D. melanogaster* Orco, initially coded as Or83b (Vosshall and Hansson, 2011), new questions arose about the role of this receptor, since it was both highly conserved across insect species and expressed in all ORN types (Vosshall et al., 2000). Nowadays, it is known that Orco and ORs are a functional heteromeric complex as odorant-sensing cation channels, which ultimately provide the rapid odorant signaling of insects, which is transient, sensitive and prolonged in nature (Neuhaus et al., 2004; Wicher et al., 2008). For comprehensive reviews around insect ORs, we recommend Yan et al. (2020) and Wicher and Miazzi (2021).

Besides the identification of Orco, the role of ORs as specific subunits was first tested in *B. mori* OR1 (BmorOR1) through electrophysiological recordings using *Xenopus* oocytes (Sakurai et al., 2004). The authors reported outstanding specificity of BmorOR1, responding to bombykol but not bombykal. Using *D. melanogaster* OR/Orco, Wicher et al. (2008) and Sato et al. (2008) were able to answer how signal transduction functions upon OR activation. Their results suggested that ionotropic responses are followed by membrane depolarization and, ultimately, metabotropic responses are unleashed. More recently, the use of CRISPR-Cas9 to disrupt the expression of Orco in the hawkmoth *M. sexta* showed that Orco is crucial for sexual communication but not for host location (Fandino et al., 2019). Despite all these advances based on model insects, the study of a non-model insect established a milestone in insect olfaction. The first three-dimensional (3D) structure of the Orco was from the fig parasitic wasp, *Apocrypta bakeri* (Butterwick et al., 2018). This has provided key knowledge on structure for these receptors. Consequently, the *A. bakeri* Orco structure has been used as a template for homology modeling on OR46 and OR49 of bark beetle *Ips typographus*, a serious threat to forest ecosystems (Yuvaraj et al., 2021).

Furthermore, the development of techniques, such as Calcium imaging through HEK293 cells (Corcoran et al., 2014), electrophysiology through *Xenopus* oocytes (Choo et al., 2018) and single-sensillum recordings (SSRs) from *Drosophila* (de Fouchier et al., 2017), have allowed functional studies for non-model insects. For instance, by using the moth *E. postvittana* the utilization and capabilities of HEK293 cells in insects was demonstrated (Corcoran et al., 2014). Another example is aphid *Acyrthosiphon pisum*, where functional assays with ORs enabled the identification of new candidate volatiles for aphid control strategies and demonstrated that these assays are suitable for aphid ORs (Zhang et al., 2017).

## Relevant Non-Model Insects for the Advance of Olfactory Physiology

In the last decade, different insect species have contributed to the understanding of peripheral olfactory systems, which today are known for being important economic pests, such as *S. littoralis* or *Helicoverpa armigera*. In our analyses, groundbreaking findings have been reported from early non-model insects, which are summarized in Table 1. As shown, the order Lepidoptera has contributed the most to olfactory physiology and, consistent with the agricultural importance that these have, we propose that these species can be considered study models. This summary supports the fact that new discoveries are difficult to be achieved based on a few insect species (e.g., study models). Thus, the particularities presented by these insects such as the inverse sexual communication of *G. mellonella* and the predatory behavior of *H. convergens* that allows the control of pests, such as aphids generate new study opportunities that model insects, such as *D. melanogaster* and *B. mori*, cannot provide. Also, the study of non-model insects shed lights to new patterns of insect biology that had not been studied before.

**TABLE 1:** Summary of early non-model insects and their contribution to olfactory physiology in the last decade.

Specie	Order	Host	Genome^a^	Contribution	References
*Galleria mellonella*	Lepidoptera	Beehives	Yes	Advance in understanding inverse sexual communication	Leyrer and Monroe (1973), Mylonakis et al. (2005), Fuchs et al. (2010), Lizana et al. (2020)
				Study model for bacterial and fungal pathogenesis	
*Spodoptera littoralis*	Lepidoptera	Cotton	No	The first comparison of adult and larval olfactory gene repertoires	Poivet et al. (2013), De Fouchier et al. (2017), Bastin-Héline et al. (2019)
				Comprehensive functional characterization of ORs	
				Novel lineage of candidate pheromone receptors	
*Dendrolimus punctatus*	Lepidoptera	Pine	Yes	A novel lineage of candidate pheromone receptors	Shen et al. (2020)
*Eriocrania semipurpurella*	Lepidoptera	Betulaceae	No	A non-ditrysian origin and evolution of pheromone receptors across Lepidoptera	Yuvaraj et al. (2017)
*Helicoverpa armigera*	Lepidoptera	Cabbage Tobacco	Yes	Sensitivity of an OR regulates optimal mating time, ensuring viability in fecundity	Chang et al. (2017), Wang et al. (2020)
				A leg-biased OBP involved in flight activity	
*Holotrichia oblita*	Coleoptera	Roots (Peanut, Soybean, Corn)	No	Co-expression of OBPs may potentiate the range of odors to which olfactory receptor neurons respond	Deng et al. (2012), Wang et al. (2013)
*Hippodamia convergens*	Coleoptera	Aphids	No	Olfactory behavior of lady beetles allows identification of prey by sex pheromone (green peach aphid)	Acar et al. (2001)
					Verheggen et al. (2007)
*Apocrypta bakeri*	Hymenoptera	*Ficus hispida*	Yes	The first three-dimensional (3D) structure of Orco	Butterwick et al. (2018)
*Manduca sexta*	Lepidoptera	Tobacco	Yes	The first complete analysis of the antennal transcriptome	Grosse-Wilde et al. (2011)
*Acyrthosiphon pisum*	Hemiptera	Alfalfa, pea, legumes	Yes	Genomic model system for ecological, developmental and evolutionary studies	Brisson and Stern (2006), Peccoud and Simon (2010)
					Vellichirammal et al. (2016)
*Dendroctonus ponderosae*	Coleoptera	Pine trees	Yes	The first functionally characterized cytochrome P450 (DponCYP345E2) in insect olfaction	Keeling et al. (2013)

a Based on NCBI Assembly and InsectGenome databases

A decade ago, advances in olfactory knowledge set a precedent in the understanding of molecular mechanisms related to insect olfaction. An example of this is the first complete analysis of the antennal transcriptome involved in olfaction in what could had been considered a non-model insect, *M. sexta* (Grosse-Wilde et al., 2011). Another example is the first comparison of adult and larval olfactory gene repertoires in the then non-model moth *S. littoralis* (Poivet et al., 2013). Currently, both Lepidopterans can be considered model insects according to the contributions in olfactory physiology and number of studies on each moth. The same happens with other insects that today can be considered study models, but a decade ago were still incipient case studies, such as *Holotrichia oblita*, *H. armigera* and *G. mellonella*.

Besides Lepidopterans, it is observed that Hemipterans appear in a large number of studies, explained by their condition as agricultural pests (Sorensen, 2009; Dedryver et al., 2010). Thus, species such as *Adelphocoris lineolatus*, *Myzus persicae*, *Sitobion avenae*, among others, can be found (Supplementary Table S2). By contrast, Hemipterans appear under-represented. An important number of studies around olfaction (i.e., OBPs, CSPs and ORs) have been performed on different species of Hemipterans, and besides *A. pisum*, we were unable to find studies that represent a breakthrough in the research field. On the other hand, the order Orthoptera includes insects that cause serious damage to crops and tree seedlings, such as leafhoppers (Joshi et al., 1999; Akhtar et al., 2012). However, no studies were found in comparison with those included in Table 1. Without underestimating evidence on Hempiterans and Orthopterans, we believe it is important to advance in future studies that can enhance our knowledge on their olfactory physiology, behavior and likely control methods.

### Role of Sequencing Techniques on Non-Model Insect Research

**FIGURE 1:**
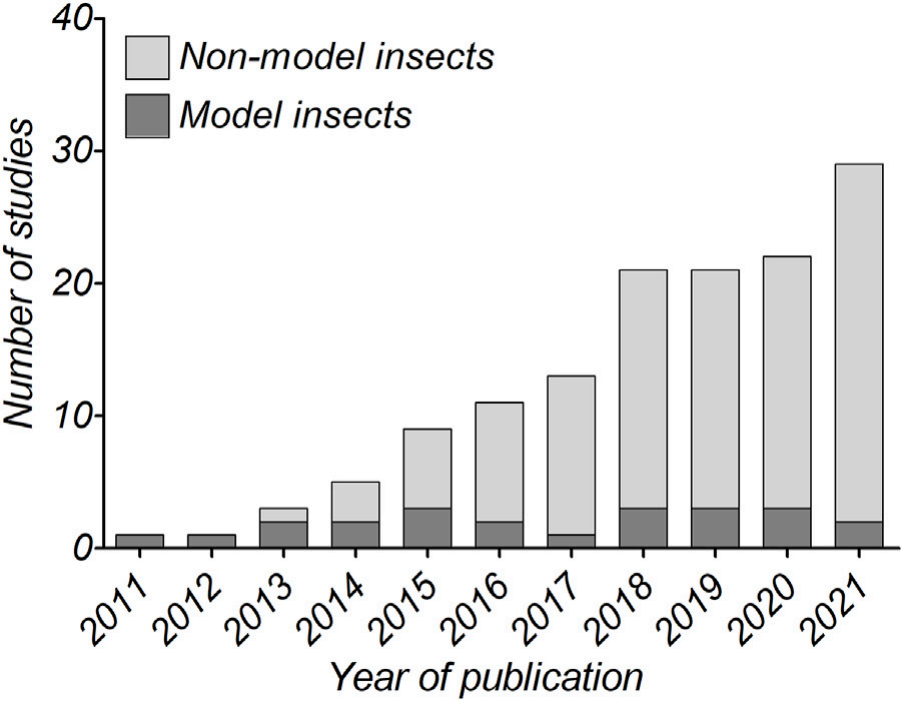
Number of studies published in the last 10 years on olfactory protein identification from antennal transcriptome (detailed data available in Supplementary Table S1).

The development of next generation sequencing (NGS) has increased both genomic and transcriptomic studies of species with limited information (Ekblom and Galindo, 2010; McCormack et al., 2013; Wachi et al., 2018). These techniques have contributed to the discovery of levels and patterns of genetic diversity, phylogenetic relationships and adaptations (Nadeau and Jiggins, 2010; Stapley et al., 2010). Examples are *D. melanogaster*, *B. mori*, *A. gambiae* and *T. castaneum* (Adams et al., 2000; Holt et al., 2002; Mita et al., 2004; Richards et al., 2008), for which genomes were deposited in the early 2000s. Furthermore, more precise and broad phylogenetic analyses have been performed. For example, the first phylogenomic tree, performed in 2014, had 2,696 genes from Lepidopterans, establishing an evolutionary framework in relation to butterflies and moths (Kawahara and Breinholt, 2014). It is worth mentioning that during the last decade, extraordinary efforts have resulted in hundreds of olfactory proteins identified through both genome and transcriptome sequencing projects. By 2019, Li et al. (2019) reported that 1,219 insect genome-sequencing projects were registered in the National Center for Biotechnology Information (NCBI). Likewise, Venthur and Zhou (2018) showed that 54 antennal or head transcriptomes (RNA-seq technology) were published. The advance in RNA-seq has allowed the expansion in type and number of samples to be sequenced. For instance, 67 RNA-seq datasets have been reported for *M. sexta* (a recent model insect), including different tissues and developmental stages (Cao and Jiang, 2017). Despite this, there are still other insect species, considered non-models as discussed above, with lack of sequencing data (Oppenheim et al., 2015). It is noteworthy that they are of great importance as agricultural pests or because they have special features that allow advances in scientific research. Nevertheless, an important increase in genomic and transcriptomic studies in specific insect species (i.e., non-model insects) has occurred in recent years (Figure 1.). Thus, it can be seen that the ratio of studies on model:non-model insects has changed from 1:1 to 1:14. Therefore, lower costs in sequencing projects, the rapid spread of insects worldwide and the search for control/monitoring methods are among those factors that can explain these findings.

Examples of how useful these techniques have become is the development of a computational acetyl cholinesterase that, together with RNA-seq, determined insecticide resistance mutations in insects like *Plutella xylostella*, *Chilo suppressalis* and *Bemisia tabaci* (Guo et al., 2017). Interestingly, CSPs and OBPs have been found to be involved in insecticide contact in *P. xylostella*, where CSP4, CSP8 and OBP13 are reported to be up-regulated after permethrin treatment (Bautista et al., 2015). Furthermore, it has been shown that the use of NGS can provide larger and more efficient large-scale RNA interference (RNAi) targets to knock-down gene of interests, allowing their application along with more conventional pest control strategies (Wang et al., 2011). As shown in Figure 1, over the years antennal transcriptome studies have steadily increased, and the identification of chemosensory proteins has gained great scope for researchers, with emphasis on non-model insects. It is believed that in addition to the large amount of data from transcriptomics, the next challenge is genomic sequencing and the subsequent collection of massive datasets for non-model species.

## Spread of Non-Model Insects: Road to be Study Models of Economic Importance

The growing and uncontrollable demographic trends have caused great concern because of an increased demand for food. Estimates have been made that by the year 2050 this demand will be up to 60% higher (Alexandratos and Bruinsma, 2012). In that sense, agriculture plays a significant role in food production (Tilman et al., 2011). Thus, it is estimated that with globalization more than one-tenth of all known pests have reached other countries where their hosts grow (Bebber et al., 2014). More discouraging is the fact that climate change can affect insect pest establishment, with increased invasion by insects due to a mixture of factors, such as changes in precipitation and increases in both atmospheric CO_2_ and temperature (Skendžić et al., 2021). On the other hand, it is proposed that a decline in flying insects has occurred in recent years due to intensive agriculture as well as urbanization and overuse of insecticides (Eggleton, 2020). However, we as well as Eggleton (2020) argue that this is a preliminary statement, and that anthropological activity (e.g., agriculture) can selectively support some insect species, such as polyphagous pests, to settle in new geographic areas. Consequently, it is crucial that in the face of a changing world, basic research on a diversity of insects can be performed. In particular, chemosensation (i.e., study of OBPs, CSPs or ORs) can provide relevant information considering it is the main driving force for insect behavior. Our literature analyses suggest a significant increase in studies related to chemosensation of non-model insects during the last decade (Table 2), from 16 to more than 80 published research articles every year. Remarkably, China is positioned as a highly productive country with 435 studies in the last decade followed by the US and France with 27 and 19, respectively. This finding could be related to the diversity of insects that have been studied. For instance, 11 different insect species were studied by 2011, whereas it scaled up to 71 in 2020 and 52 in 2021. Despite this diversity, there are some insects that were not initially considered as models and that have been systematically studied in the last 10 years, such as *S. littoralis*, *A. lineolatus* and *H. armigera*. It is worth mentioning that about 20 studies related to olfaction of each of these species have been published. Hence, these insects can be considered as models for the number of studies in the area. Finally, this summary evidences the impact of agricultural pests in the last few years and how research teams have had to address their control and monitoring.

**TABLE 2:** Summary of insect olfaction studies in the last decade.

Year	Geographical area^a^ ^,^ ^b^	Total number of studies	Number of insect species studied	Most studied insect^b^
2011	China (8); Canada (2); EE.UU. (3); France (2); Germany (1)	16	11	*Manduca sexta* (2); *Spodoptera littoralis* (2); *Locusta migratoria* (2); *Heliothis virescens* (2); *Adelphocoris lineolatus* (2)
2012	EE.UU. (6); China (24); Italy (2); France (4); New Zealand (2)	37	32	*Spodoptera littoralis* (4); *Helicoverpa armigera* (4)
2013	China (28); EE.UU. (5); India (1); France (2); Germany (1); Sweden (2); Mexico (1)	40	23	*Helicoverpa armigera* (5)
2014	EE.UU. (3); China (31); Brazil (1); Chile (1); Italy (1); Belgium (1); Australia (1); Germany (2); France (2); Sweden (1); Norway (1)	43	36	*Spodoptera exigua* (4)
2015	China (35); EE.UU. (2); Sweden (1); France (1); New Zealand (2); Australia (1); Japan (1); India (1); Thailand (1); Brazil (1)	47	37	*Spodoptera litura* (6)
2016	China (42); EE.UU. (2); Brazil (1); India (1); Saudi Arabia (2); United Kingdom (1); Sweden (2); France (1); Japan (3); Chile (1); Italy (1); Canada (1)	58	52	*Cydia pomonella* (4)
2017	Germany (3); China (45); France (1); Sweden (1); Brazil (1); EE.UU. (3)	54	44	*Adelphocoris lineolatus* (5)
2018	Sweden (2); Chile (1); China (53); Japan (1); Saudi Arabia (1); Italy (1); EE.UU. (1); Canada (1); India (1); France (1)	63	53	*Bactrocera dorsalis* (3); *Grapholita molesta* (3)
2019	China (51); Chile (3); Taiwan (1); Sweden (4); Germany (3); Italy (2); EE.UU. (1)	65	57	*Bactrocera minax* (3); *Spodoptera littoralis* (3)
2020	China (71); Chile (2); Mexico (2); Canada (1); United Kingdom (1); Sweden (1); France (1); Germany (1); Spain (1); Kenya (1); EE.UU. (1)	83	71	*Bactrocera minax* (4)
2021	China (47); EE.UU. (1); Panama (1); Saudi Arabia (2); Italy (2); Austria (1); Australia (1); Kenya (1); France (4); Sweden (2); Colombia (1)	63	52	*Adelphocoris lineolatus* (3); *Rhynchophorus ferrugineus* (3); *Spodoptera littoralis* (3)

a Countries have been showed in no particular order.

b Number in parenthesis indicates amount of published studies that year. Full database can be found in Supplementary Table S2.

Among those insects as study models, *D. melanogaster* can be considered the most famous “workhorse” for bioinformatics, experimental biology and genetics, with 6 Nobel prizes to its credit. However, *D. melanogaster* is a small representative of a large lineage of *Drosophila* species. An interesting subgroup of species from *melanogaster* is the *suzukii* subgroup, where *D. suzukii* appears. This fly is a serious pest of berries, laying eggs in healthy unwounded fruits (Sasaki and Sato, 1995). Originally linked to Southeast Asia, particularly Japan, China, Taiwan, North and South Korea, and even the Russian Far East, *D. suzukii* has spread rapidly across the European and American continents (Cini et al., 2012). Nowadays, it can be found in South America and Africa (Deprá et al., 2014; Hassani et al., 2020). Interestingly, studies around chemosensation in *D. suzukii* are only recent compared with other insect species mentioned in this review. For instance, 71 OR genes were identified in 2016 by comparative genomics, including *D. suzukii*, *D. biarmipes* and *D. takahashii* (Hickner et al., 2016). More recently, the differential expression of ORs was evaluated, which appeared to be modulated by post-mating status in *D. suzukii* females (Crava et al., 2019). Likewise, 27 chemosensory genes, such as ORs, OBPs and CSPs, were found to be sex-biased in expression by 2020 (Ahn et al., 2020). Recent evidence in 2021 reported the role of OBP69a and OBP76a in *D. suzukii*, being functional against floral compounds, such as β-ionone (Zhan et al., 2021). These advances represent an important first step towards likely big discoveries around the chemical ecology of *D. suzukii*, which could become a study model in the near future.

Another example of a non-model insect that has spread in recent years is the grapevine moth *Lobesia botrana*. This moth is reported as the most harmful pest in grapevine production in Southern and Central European countries (Moschos et al., 2004), and currently, including South and North American countries, such as the wine-growing regions of Chile, California and *Argentina*. The first report of *L. botrana* was in Austria in 1776 by Denis and Schiffermüller; however, its geographic origin remains uncertain. Although this moth is linked to grapes and originally to berries from *Daphne gnidium*, it is considered a polyphagous insect (Maher and Thiéry, 2006). Given its rapid spread and economic importance, it has been extensively studied in terms of its chemical ecology and pest management (Tasin et al., 2010; Chelef et al., 2020). In relation to chemosensation, recent studies have addressed the identification and functional characterization of olfactory proteins. Thus, a profile of 61 ORs, 35 OBPs and 18 CSPs was reported for *L. botrana* in 2018 (Rojas et al., 2018), and an OBP tuned to pheromone components was reported in 2019 (Venthur and Xhou, 2018). It is believed that *L. botrana*, like other Tortricids of economic importance, such as *C. pomonella* and *G. molesta*, will likely become a study model in the future.

Besides Dipterans and Lepidopterans, Coleopterans have been a matter of research in recent years. An example is the red palm weevil *Rhynchophorus ferrugineus*, which attacks palm trees in a mass coordinated process (Oehlschlager, 2016). Originally related to South and Southeast Asia, this insect has reached worldwide distribution (El-Mergawy and Al-Ajlan, 2011; Fouda et al., 2022). In terms of olfaction, important advances have been made in recent years. For example, the OR, RferOR1, has been identified as active against the pheromone ferrugineol ((4*RS*,5*RS*)-4-methylnonan-5-ol) and ferrugineone ((4*RS*)-methylnonan-5-one) (Antony et al., 2016). Likewise, the profile of olfactory proteins (e.g., 37 OBPs, 10 CSPs and 63 ORs, among others) has been reported (Gonzalez et al., 2021). In fact, the genome of *R. ferrugineus* has been published, suggesting that this insect will become a study model (Hazzouri et al., 2020). Another example are bark beetles, such as Ips typographus, an important forest pest, where olfactory proteins (15 OBPs, 6 CSPs, 3 SNMPs, 43 ORs, 6 GRs and 7 IRs) have been identified and analyzed (Andersson et al., 2013). Recently, putative ligand binding sites have been identified in ORs of this bark beetle that affect the responses of these insect receptors (Yuvaraj et al., 2021).

Other insect species, such as aphids *M. persicae* and *S. avenae* as well as Lepidopterans related to stored food pests, such as *Plodia interpunctella*, are already being studied according to our literature analysis (Supplementary Table S2). Thus, depending on their contribution to insect chemosensation, we argue that these insects (as well as others) can become study models. Nevertheless, we believe that each contribution strengthens the study of olfactory physiology, and pushes the limits towards more in-depth research. Thus, in addition to the economic importance of these insects, over the years the gap between model and non-model insects has been narrowing, leading to an increase in knowledge at the chemosensory level. However, there are still insects that have not been studied or have been little studied. An example is *Euborellia annulipes*, an important predator of pest insects, whose olfactory system has not yet been studied (da Silva Nunes et al., 2020; Tangkawanit et al., 2021).

## Control of Non-Model Insects Still Led by Conventional Integrated Pest Management Strategies

With industrial revolution pushing the limits of productive areas, agriculture was no exception. By 1940 insect pest control strategies were needed to mitigate negative impacts; therefore, insects were controlled using man-made chemicals, called pesticides (Jones, 1998). However, concerns were raised in the ensuing decades because of pesticide persistence, groundwater contamination and appearance of resistance in insects (Hou and Wu, 2010; Hillocks, 2012; Garrigou et al., 2020). Therefore, alternative approaches emerged, such as the use of semiochemicals, which served for integrated pest management (IPM) strategies. This term is defined as a pest management strategy that employs methods consistent with economic, ecological and toxicological requirements in order to maintain pests below the economic threshold, giving priority to natural limiting factors (Katsoyannos, 1992; Jones, 1998). Consequently, it was necessary to understand the biology and life cycles of insect pests to apply IPM strategies. In that sense, several olfaction-dependent techniques raised in the context of IPM strategies, such as mating disruption, mass trapping, lure and kill and push-pull (Jones, 1998). For a more in-depth review of these techniques, we suggest readers to check Nesreen and Abd El-Ghany (2019), and Morrison et al. (2021).

During the 2000s, the first steps in understanding insect olfactory systems allowed some advances in the area of insect pest control, where OBPs were used as targets, despite being discovered years before by Vogt and Riddiford (1981). Thus, OBPs were the first target to use to clarify behaviorally active chemicals as a response to issues in semiochemically-based IPM strategies, such as time-consuming experiments for semiochemical identification, extensive bioassays at laboratory and field level, and related costs. Nowadays, OBPs and ORs have been proposed to be key targets for pest control, thus ligand binding to these proteins could interfere with specific insect behaviors (Venthur and Zhou, 2018). Therefore, the last decade has been marked by the development of reverse chemical ecology approaches, which take advantage of olfactory proteins (i.e., OBPs or ORs) to identify novel potent semiochemicals for pest control (Leal, 2005). The first successful study was on the OBP1 of mosquito *C. quinquefasciatus*, where trimethylamine and nonanal exhibited potent attraction (Leal et al., 2008). More recently, OR36 of *C. quinquefasciatus* has been studied, which from a panel of 230 odorants, acetaldehyde was the strongest in activating OR36 (Choo et al., 2018). Likewise, 4-methylcyclohexanol was found as a specific activator of OR80 in Chagas vector *Rhodnius prolixus*, being repellent in behavioral assays (Franco et al., 2018). In addition, with scientific advances in the area, reverse chemical ecology has been implemented in non-model insects of agricultural importance. In the oriental fruit moth *G. molesta*, it was demonstrated through behavioral and field trapping assays that the compound codlemone ((*E*,*E*)-8,10-dodecadienol) is an excellent pheromone synergist that can be detected by GmolPBP2 and also provides an optimization of commercial sexual attractants used in the control of this pest (Liu et al., 2022).

It is worth noting that in recent years reverse chemical ecology has evolved into a genetically-based approach. For example, in the cotton leafworm *S. littoralis,* the CRISPR-Cas9 technique was used to edit the genome by eliminating the Orco gene (Koutroumpa et al., 2016). Results showed that more than 80% of the individuals had mutations in the Orco gene, passing them on to the next generation, which caused problems in the olfactory detection of pheromones by this moth. This appears to be the first step into new, ethically debatable, control techniques for specific species.

Despite the progress in olfactory physiology in both model and non-model insects, the jump towards IPM strategies based on OBPs, CSPs and/or ORs, remains elusive. Currently, many insect pests are still monitored and/or controlled by conventional IPM strategies. An example is the use of pheromones, which have proven to be a successful method (i.e., mating disruption) for several pest species, mainly in Lepidoptera (Conchou et al., 2019), by releasing high concentrations of pheromones in crop fields (Reddy and Guerrero, 2010; Benelli et al., 2019). Recently, sex pheromone-baited traps ((*Z*,*Z*)-7,10-hexadecadienal) were designed for monitoring the apple orchard pest *Chilecomadia valdiviana*, certainly a non-model insect (Barros-Parada et al., 2021). The mating disruption technique has also been used against the olive moth *Prays oleae* to suppress damage in olives (Ortiz et al., 2021). The authors’ findings suggest that aerosol-type traps could yield >75% of male captures and 20% less damage in olives.

Other types of conventional IPM techniques are also still used. For example, the push-pull strategy is a technique that repels an insect pest from a crop of interest, while attracting it towards an external location. A reduction in population of leafhopper *Empoasca flavescens* has been reported by using repellents and attractants to control the insect (Niu et al., 2022). Likewise, mass trapping has been useful in controlling medfly *Ceratitis capitata*, which functions as powerful trap full of attractants (Hafsi et al., 2019). For *C. capitata*, two food attractants (amine and organic acids) were released from baited sprays, which resulted in low damage (2.2%–3.9%) to citrus fruits.

To date, it is clear that IPM strategies are pivotal in insect pest control and monitoring in order to pursue environmentally friendly methods. However, with the enormous number of olfactory proteins identified year by year in response to the spread of non-model insects, we strongly believe that significant discoveries and technological advances are yet to come.

## Concluding Remarks

The study of insect olfaction is no longer a novel approach for integrated pest management. It has evolved from the study of a few insect species and their olfactory proteins to dozens of insect species and several type of proteins. This might be led by such factors as insecticide resistance, intensive agriculture and climate change that have affected spread and establishment of pests worldwide. Consequently, sequencing technologies are becoming increasingly feasible for studying insect olfaction. This has resulted in groundbreaking evolutionary studies as well as improved integrated pest management strategies. Particularly, non-model insects have provided us with a profound knowledge of olfaction, from structural features of receptors (*A. bakeri* Orco) to tissue-biased expression with special functions (*H. armigera* OBP3). Furthermore, we believe that the study of non-model insects, in addition to providing multiple benefits for agriculture, can pave the way to face future challenges in a changing ecosystem.
