# Supplementary material for: Insights Into Chemosensory Proteins From Non-Model Insects: Advances and Perspectives in the Context of Pest Management
Source: Front Physiol. 2022 Aug 22;13:924750. doi: 10.3389/fphys.2022.924750 (PMC9441497; doi:10.3389/fphys.2022.924750)
Supplement: Supplementary file 2 [file Table1.docx]

**Supplementary Table 1.** Studies performed on the identification of olfactory proteins from the antennal transcriptome.

| **Insect species** | **Insect order** | **Model or Non-model*** | **References** |
| --- | --- | --- | --- |
| *Blattella germanica* | Blatodea | Non-model | Niu et al., 2016 |
| *Aethina tumida* | Coleoptera | Non-model | Cilia & Nanetti, 2021 |
| *Rhynchophorus ferrugineus* | Coleoptera | Non-model | Rasool et al., 2021 |
| *Agrilus zanthoxylumi* | Coleoptera | Non-model | Gong et al., 2020 |
| *Anomala corpulenta* | Coleoptera | Non-model | Chen et al., 2014 |
| *Anoplophora chinensi* | Coleoptera | Non-model | Wang et al., 2017 |
| *Anoplophora glabripennis* | Coleoptera | Non-model | Hu et al., 2017 |
| *Apolygus lucorum* | Coleoptera | Non-model | Zheng et al., 2020 |
| *Apriona germari* | Coleoptera | Non-model | Qian et al., 2020 |
| *Batocera horsfieldi* | Coleoptera | Non-model | Hu et al., 2019 |
| *Callosobruchus chinensis* | Coleoptera | Non-model | Zhang et al., 2017 |
| *Coccinella septempunctata* | Coleoptera | Non-model | Yang et al., 2020 |
| *Colaphellus bowringi* | Coleoptera | Non-model | Li et al., 2015 |
| *Cylas formicarius* | Coleoptera | Non-model | Bin et al., 2017 |
| *Dendroctonus valens* | Coleoptera | Non-model | Gu et al., 2015 |
| *Eucryptorrhynchus brandti* | Coleoptera | Non-model | Wen et al., 2018 |
| *Eucryptorrhynchus scrobiculatus* | Coleoptera | Non-model | Wen et al., 2018 |
| *Harmonia axyridis* | Coleoptera | Non-model | Rondoni et al., 2021 |
| *Holotrichia parallela* | Coleoptera | Model | Yi et al., 2018 |
| *Hylamorpha elegans* | Coleoptera | Non-model | Gonzalez-Gonzalez et al., 2019 |
| *Leptinotarsa decemlineata* | Coleoptera | Non-model | Liu et al., 2015 |
| *Lissorhoptrus oryzophilus* | Coleoptera | Non-model | Zhang et al., 2019 |
| *Ophraella communa* | Coleoptera | Non-model | Ma et al., 2019 |
| *Photinus pyralis* | Coleoptera | Non-model | Tran, 2020 |
| *Protaetia brevitarsis* | Coleoptera | Non-model | Liu et al., 2019 |
| *Rynchophorus ferrugineus* | Coleoptera | Non-model | Antony et al., 2016 |
| *Semanotus bifasciatus* | Coleoptera | Non-model | Zhang et al., 2019 |
| *Sitophilus zeamais* | Coleoptera | Non-model | Tang et al., 2019 |
| *Tenebrio molitor* | Coleoptera | Model | Liu et al., 2015 |
| *Tomicus yunnanensis* | Coleoptera | Non-model | Liu et al., 2018 |
| *Anopheles gambiae* | Diptera | Model | Rinker et al., 2013 |
| *Haematobia irritans* | Diptera | Non-model | Olafson & Sask, 2020 |
| *Procecidochares utilis* | Diptera | Non-model | Li et al., 2020 |
| *Aldrichina graham* | Diptera | Non-model | Han et al., 2020 |
| *Bactrocera dorsalis* | Diptera | Non-model | Liu et al., 2020 |
| *Bactrocera minax* | Diptera | Non-model | Xu et al., 2019 |
| *Bradysia odoriphaga* | Diptera | Non-model | Zhao et al., 2018 |
| *Calliphora stygia* | Diptera | Non-model | Leitch et al., 2015 |
| *Drosophila suzukii* | Diptera | Model | Crava et al., 2019 |
| *Drosophila melanogaster* | Diptera | Model | Shiao et al., 2013 |
| *Episyrphus balteatus* | Diptera | Non-model | Wang et al., 2017 |
| *Hermetia illucens* | Diptera | Non-model | Xu et al., 2020 |
| *Propsilocerus akamusi* | Diptera | Non-model | Yan et al., 2020 |
| *Corythucha ciliata* | Hemiptera | Non-model | Yang et al., 2021 |
| *Leptocorisa acuta* | Hemiptera | Non-model | Qu et al., 2020 |
| *Rhopalosiphum padi* | Hemiptera | Non-model | Kang et al., 2018 |
| *Adelphocoris lineolatus* | Hemiptera | Non-model | Xiao et al., 2017 |
| *Adelphocoris suturalis* | Hemiptera | Non-model | Cui et al., 2017 |
| *Arma chinensis* | Hemiptera | Non-model | Wu et al., 2020 |
| **Insect species** | **Insect order** | **Model or Non-model** | **References** |
| *Cacopsylla chinensis* | Hemiptera | Non-model | Xu et al., 2019 |
| *Cyrtorhinus lividipennis* | Hemiptera | Non-model | Wang et al., 2018 |
| *Empoasca onukii* | Hemiptera | Non-model | Bian et al., 2018 |
| *Halyomorpha halys* | Hemiptera | Non-model | Paula et al., 2016 |
| *Laodelphax striatellus* | Hemiptera | Non-model | Li et al., 2020 |
| *Nezara viridula* | Hemiptera | Non-model | Wu et al., 2019 |
| *Nilaparvata lugens* | Hemiptera | Non-model | Zhou et al., 2014 |
| *Phenacoccus solenopsis* | Hemiptera | Non-model | Nie et al., 2018 |
| *Sitobion avenae* | Hemiptera | Non-model | Xue et al., 2016 |
| *Subpsaltria yangi* | Hemiptera | Non-model | Qi et al., 2018 |
| *Tropidothorax elegans* | Hemiptera | Non-model | Song et al., 2018 |
| *Yemma signatus* | Hemiptera | Non-model | Song et al., 2021 |
| *Bemisia tabaci* | Homoptera | Non-model | Wang et al., 2017 |
| *Aphidius gifuensis* | Hymenoptera | Non-model | Kang et al., 2021 |
| *Aulacocentrum confusum* | Hymenoptera | Non-model | Li et al., 2021 |
| *Crematogaster rogenhoferi* | Hymenoptera | Non-model | Zhou et al., 2021 |
| *Aenasius bambawalei* | Hymenoptera | Non-model | Nie et al., 2018 |
| *Apis cerana cerana* | Hymenoptera | Model | Peng et al., 2017 |
| *Chouioia cunea* | Hymenoptera | Non-model | Zhao et al., 2016 |
| *Cotesia vestalis* | Hymenoptera | Non-model | Liu et al., 2020 |
| *Encarsia formosa* | Hymenoptera | Non-model | He et al., 2020 |
| *Osmia cornuta* | Hymenoptera | Non-model | Yin et al., 2013 |
| *Sirex nitobei* | Hymenoptera | Non-model | Guo et al., 2021 |
| *Sirex noctilio* | Hymenoptera | Non-model | Guo et al., 2021 |
| *Trichogramma chilonis* | Hymenoptera | Non-model | Liu et al., 2018 |
| *Achelura yunnanensis* | Lepidoptera | Non-model | Li et al., 2021 |
| *Agrotis ipsilon* | Lepidoptera | Model | Gu et al., 2014 |
| *Athetis dissimilis* | Lepidoptera | Non-model | Sun et al., 2016 |
| *Carposina sasakii* | Lepidoptera | Non-model | Tian et al., 2018 |
| *Chilo suppressalis* | Lepidoptera | Model | Cao et al., 2014 |
| *Clostera restitura* | Lepidoptera | Non-model | Gu et al., 2019 |
| *Conogethes pinicolalis* | Lepidoptera | Non-model | Jing et al., 2020 |
| *Conogethes punctiferalis* | Lepidoptera | Model | Jia et al., 2016 |
| *Cydia pomonella* | Lepidoptera | Model | Bengtsson et al., 2012 |
| *Cydia fagiglandana* | Lepidoptera | Non-model | Gonzalez et al., 2017 |
| *Cydia nigricana* | Lepidoptera | Non-model | Gonzalez et al., 2017 |
| *Dendrolimus kikuchii* | Lepidoptera | Non-model | Zhang et al., 2014 |
| *Dendrolimus houi* | Lepidoptera | Non-model | Zhang et al., 2014 |
| *Dioryctria abietella* | Lepidoptera | Non-model | Xing et al., 2021 |
| *Eogystia hippophaecolus* | Lepidoptera | Non-model | Hu et al., 2016 |
| *Eupeodes corollae* | Lepidoptera | Non-model | Yuvaraj et al., 2017 |
| *Galleria mellonella* | Lepidoptera | Model | Zhao et al., 2019; Lizana et al., 2020 |
| *Grapholia molesta* | Lepidoptera | Non-model | Li et al., 2015b |
| *Helicoverpa assulta* | Lepidoptera | Model | Zhang et al., 2015a |
| *Hedya nubiferana* | Lepidoptera | Non-model | Gonzalez et al., 2017 |
| *Helicoverpa armigera* | Lepidoptera | Model | Zhang et al., 2015 |
| *Heortia vitessoides* | Lepidoptera | Non-model | Cheng et al., 2019 |
| *Histia rhodope* | Lepidoptera | Non-model | Yang et al., 2020 |
| *Hyphantria cunea* | Lepidoptera | Non-model | Zhang et al., 2016 |
| *Lobesia botrana* | Lepidoptera | Model | Rojas et al., 2018 |
| *Manduca sexta* | Lepidoptera | Model | Grosse-Wilde et al., 2011 |
| *Mythimna separata* | Lepidoptera | Model | Chang et al., 2017 |
| *Oraesia emarginata* | Lepidoptera | Non-model | Feng et al., 2017 |
| **Insect species** | **Insect order** | **Model or Non-model*** | **References** |
| *Ostrinia furnacalis* | Lepidoptera | Model | Zhang et al., 2015 |
| *Pieris rapae* | Lepidoptera | Model | Li et al., 2020; Yang et al., 2021 |
| *Plodia interpunctella* | Lepidoptera | Non-model | Jia et al., 2018 |
| *Semiothisa cinerearia* | Lepidoptera | Non-model | Liu et al., 2020 |
| *Spodoptera exigua* | Lepidoptera | Non-model | Du et al., 2018 |
| *Spodoptera frugiperda* | Lepidoptera | Non-model | Qiu et al., 2020 |
| *Spodoptera littoralis* | Lepidoptera | Model | Walker et al., 2019 |
| *Spodoptera litura* | Lepidoptera | Model | Feng et al., 2015 |
| *Streltzoviella insularis* | Lepidoptera | Non-model | Yang et al., 2019 |
| *Ceracris kiangsu* | Orthoptera | Non-model | Li et al., 2020 |
| *Ceracris nigricornis* | Orthoptera | Non-model | Yuan et al., 2019 |
| *Oedaleus asiaticus* | Orthoptera | Non-model | Zhout et al., 2019 |
| *Oxya chinensis* | Orthoptera | Non-model | Cui et al., 2019 |
| *Schistocerca gregaria* | Orthoptera | Non-model | Pregitzer et al., 2017 |
| *Eriocrania semipurpurella* | Trichoptera | Non-model | Yuvaraj et al., 2018 |
| *Lampronia capitella* | Trichoptera | Non-model | Yuvaraj et al., 2018 |
| *Rhyacophila nubila* | Trichoptera | Non-model | Yuvaraj et al., 2018 |
| *Classification according to the benchmark proposed in this review. | | | |
